# A systematic review and meta-analysis of venous thromboembolism risk in surgical patients with recent air travel

**DOI:** 10.1177/02683555251342912

**Published:** 2025-05-14

**Authors:** Jessie Shea, Avik Ghosh, Benedict RH Turner, Alun H Davies, Sarah Onida

**Affiliations:** 1 Department of Trauma and Orthopaedics, 4262 Kingston Hospital NHS Foundation Trust, Kingston upon Thames, UK; 2 Department of Acute Medicine, 9820 Nottingham University Hospitals NHS Trust, Nottingham, UK; 3 Section of Vascular Surgery, Department of Surgery and Cancer, 4615 Imperial College London, London, UK

**Keywords:** Venous thromboembolism, venous thrombosis, air travel, surgery, risk

## Abstract

**Objective:**

Risk of venous thromboembolism (VTE) is classically associated with recent surgery; additionally, long-haul air travel is a known VTE risk factor. This meta-analysis aimed to estimate the post-operative VTE risk associated with recent air travel.

**Methods:**

Embase, Medline, Cochrane and Scopus databases were accessed from inception to May 2024. Inclusion criteria were any study design of participants undergoing surgical intervention with recent air travel, reporting VTE incidence. Papers were screened and data extracted independently by two reviewers, then pooled using fixed and random effects. The primary outcome was pooled VTE rate, with secondary outcomes of pooled rate of deep vein thrombosis (DVT) and pulmonary embolism (PE). Subgroup analyses of pre- and post-operative flight, flight >4 h and high VTE risk surgery were conducted. The ROBINS-I tool was used to assess risk of bias.

**Results:**

Seven retrospective studies were included in the analysis, totalling 24,975 patients. The pooled VTE odds ratio (OR) in the flying plus surgery group was 1.96 (95% CI: 0.54–7.08). For surgery with post-operative flight, the VTE OR was 1.31 (95% CI: 0.63–2.71), whilst for surgery with pre-operative flight the OR was 7.86 (95% CI: 0.23–265.26). In a subgroup analysis of air travel >4 h, the VTE OR was 2.35 (95% CI: 0.29–19.36). In the subgroup analysis of high VTE risk surgery, the VTE OR was 1.20 (95% CI: 0.45–3.20). Three studies reported DVT/PE incidence specifically. For surgery and recent air travel, the pooled DVT rate was 0.67% (95% CI: 0.31%–1.51%) versus 0.45% (95% CI: 0.10%–2.00%) in surgery alone. For surgery and recent air travel, the pooled PE rate was 0.41 (95% CI: 0.00%–1.29%) versus 0.55% (95% CI: 0.31%–0.86%) for surgery alone.

**Conclusion:**

This meta-analysis suggests that air travel confers no additional VTE risk for patients undergoing surgical intervention. However, this does not account for confounding factors. Future research should risk score then propensity match participants to generate higher quality evidence.

## Introduction

Surgery is a well established major risk factor for venous thromboembolism (VTE), with reported incidence rates of 1% across all surgical interventions.^1–4^ This confers a significant mortality risk, with an estimated 10–30% of patients dying within 30 days of VTE.^5^ Long-term sequelae are also prevalent, with both deep venous thrombosis (DVT) and pulmonary embolism (PE) associated with increased risk of recurrent VTE^6^ and persistent morbidity in the form of post-thrombotic syndrome^7^ and pulmonary hypertension.^8^ These contribute to the economic burden of VTE, with US healthcare costs for newly diagnosed and treated VTE estimated at $7–10 billion per annum.^9^ Subsequently, it is now common practice for thromboprophylaxis to be given to this group of patients following risk assessment.^10–12^ However, despite efforts, VTE remains a significant health concern, with fatal PE remaining the most common cause of preventable death in post-operative patients.^13,14^

One further risk factor independently linked to VTE is air travel. Many studies have demonstrated a positive correlation with long-haul flight and VTE,^15,16^ with reported rates of 0–2% in low-risk patients, rising to 5% in individuals with other concurrent risk factors.^17^ This elevated VTE incidence is thought to increment further with increased air travel exposure, with reported incidence rates ratios increasing by 1.1-fold for each extra hour of flight.^15^ It is postulated that venous stasis from prolonged periods of immobility, hypoxia and dehydration all contribute to this increased risk.^18^ To mitigate this, there is growing use of individualized risk assessment; mechanical thromboprophylaxis such as graduated compression stockings and/or chemical thromboprophylaxis.^19,20^

With the rise in health tourism and accessibility of air travel, it has become increasingly common for patients to fly long distances seeking surgical intervention.^21^ A 2017 estimate puts the total number of medical tourists at 1.4 million for US citizens alone, a 1.9-fold increase from 10 years previously.^22^ Colloquially, whilst many patients are aware of elevated VTE risk after flying, there are currently few resources to inform patients or guide practice as to the additional risk of peri-operative air travel. This systematic review and meta-analysis aimed to estimate the rate of post-operative VTE in the context of recent air travel from pooled published data and assessed the impact of any additional risk prolonged flying may pose.

## Methods

Preferred Reporting Items for Systematic Reviews and Meta-Analysis (PRISMA) guidance was followed in construction of this systematic review and meta-analysis.^23,24^ The protocol was pre-registered and is freely accessible (PROSPERO:CRD42022323561).

### Search strategy

Embase, Medline, Cochrane and Scopus databases were accessed from inception to 1st May 2024. The literature was searched without limitations on study design or cohort size. Searches consisted of 36 different terms covering aviation, thromboembolism, vein thrombosis, surgery, venous surgery and their appropriate Medical Subheading (MESH) terms (Supplemental Table 1).

### Eligibility criteria

• Participants undergoing surgical intervention
• Reported rate of symptomatic DVT, PE or VTE
• Peri-operative air travel (within 6 weeks of surgery)

### Exclusion criteria

• Studies not investigating the combined risk of flying and surgery
• Other forms of travel (land, sea)
• Prior known VTE risk factors in addition to surgery (pregnancy, malignancy, heritable thrombophilia)
• Articles not in English, non-human studies, conference abstracts with no full-text publication, single case reports, narrative reviews, duplicate publications

### Article screening

Articles were screened independently in accordance with the eligibility and exclusion criteria by two separate reviewers (JS, AG), any discrepancy was mediated and resolved by a third reviewer (BRHT). Title and abstract screening were conducted on EndNote X20 (Clarivate, USA) to exclude non-relevant studies before full-text review of the remaining papers.

### Data extraction

Following full-text screening, data was extracted independently by two reviewers (JS, AG) using a pre-specified template in Microsoft Excel (Microsoft, Richmond, USA). Participant characteristics, available flight data, surgical characteristics and thromboprophylaxis data were collected, as well as rates of DVT, PE and VTE-related mortality. If data for a particular outcome was unavailable for extraction, study authors were contacted by email. If there was no response or the requested information was not recorded, this data point was noted as ‘not documented’ under the appropriate heading in the results section. Statistical analyses were computed using R v3.2.3 (R Core Team, Vienna, Austria), RStudio v1.0.44 (RStudio Inc., Boston, USA) and RevMan5 v5.4 (Cochrane Collaboration, London, UK). Where data from two-armed trials were available and heterogeneity was sufficiently low, they were pooled with fixed-effects via head-to-head meta-analysis. High heterogeneity of results (>70%) were discussed by three reviewers (JS, AG, BRHT) and resulted in a narrative synthesis alongside meta-analysis. Where data were collected from single-study arms for the rates of VTE, results were pooled using a random-effects model and meta-proportions analysis. Where overall proportions were low, a logit transformation was used to normalise the data, and where there were high proportions of zero-event rates, double arcsine-Tukey transformation was performed.

### Risk of bias and quality assessment

Risk of bias was assessed using the Risk of Bias in Non-Randomised Studies of Interventions (ROBINS-I) tool and data quality was analysed using the Grading of Recommendations Assessment, Development and Evaluation (GRADE) framework. These were undertaken by two independent reviewers (JS, AG) and any disputes resolved by a third reviewer (BRHT).

## Results

The initial search yielded 1017 papers, of which 91 were selected for full-text screening. From full-text screening, seven papers were identified for final inclusion in the analysis (Supplemental Figure 1). Of these, there were six retrospective cohort studies^25–30^ and one case-control study,^31^ with a combined total of 24,975 patients (Table 1).^25–31^ Baseline characteristics showed a slight propensity to male sex in patients identified with VTE, with a pooled ratio of 148:123.^26,28–31^ A wide distribution of patient ages were included, from 14 to 92 years old.^25–31^ Where documented, mean age ranged from 40 to 70 years.^26,27,29,31^ Only one paper commented on the age difference between the flying and non-flying groups,^29^ with the mean age of surgical patients with air travel being 18 years lower than those without (46 vs 64 years).

**Table 1.** Demographic data for included papers assessing the incidence and risk of venous thromboembolism in surgical patients with recent air travel.

Paper	Study design	Number of patients	VTE risk factors accounted for	Surgery type	Use of compression	Use of anti-coagulation	Flight duration	Flight timing	Interval between flying and surgery
Ball 2007	Retrospective cohort study	608, 462 with recent air travel	Previous VTE, family history of VTE, BMI, hormone therapy, malignancy, INR level	Elective lower limb orthopaedic (THA, resurfacing arthroplasty, revision arthroplasty)	Yes	Yes	Range of 1–12 h, mean of 4 h	Post-operatively	<42 days
Cassivi 2017	Retrospective cohort study	817, 96 with recent air travel	Not documented	Elective cardiothoracic (pulmonary resection)	Not documented	Not documented	Range of 486 -9684 km, median of 1783 km	Post-operatively	Range of 0 – 45 days
Citak 2015	Retrospective cohort study	342, 155 with recent air travel	BMI, age, gender, type of surgery	Lower limb orthopaedic (THA, TKA)	Not documented	Yes	>4 h, range not documented	Pre-operatively	Not documented
Cooper 2014	Retrospective cohort study	1465, 220 with recent air travel	BMI, age	Elective lower limb orthopaedic (THA, TKA)	Not documented	Yes	Range of 1.1 – 13.7 h, mean of 2.7 h	Post-operatively	Range of 1–10 days
Gajic 2005	Retrospective cohort study	8860, with recent air travel	ASA grade, type of surgery, VTE prophylaxis, smoking, BMI, malignancy, thrombophilia, previous VTE	Elective head & neck, thoracic & vascular, abdominal	Not documented	Yes	>5000 km, range not documented	Pre-operatively	Not documented
Kuipers 2014	Nested case control study	7142, 2060 with recent air travel	Pregnancy, hormone therapy, malignancy, plaster casting	Not documented	Not documented	Not documented	>4 h, range not documented	Post-operatively	Not documented
Mahmood 2022	Retrospective cohort study	5741, 243 with recent air travel	BMI	Elective lower limb orthopaedic (THA, TKA)	Yes	Yes	40 – 85 min, mean of 74 min	Post-operatively	Range of 1 – 24 days

Abbreviations List: ASA, American society of anaesthesiologists physical status classification system; BMI, body mass index; INR, international normalised ratio; THA, total hip arthroplasty; TKA, total knee arthroplasty; VTE, venous thromboembolism.

The majority of studies attempted to account for a number of confounding factors predisposing patients to VTE.^25,27–31^ Body Mass Index (BMI) was most commonly controlled for,^25,27–30^ followed by malignancy,^25,29,31^ age,^27,28^ hormone therapy,^25,31^ surgery type^27,29^ and previous VTE.^25,29^ In the majority of papers surgery type was elective only,^25,26,28–30^ with four papers looking exclusively at lower limb orthopaedic interventions, specifically total hip arthroplasty (THA) and total knee arthroplasty (TKA).^25,27,28,30^ Other operations included were pulmonary resections and elective head and neck, vascular and abdominal surgery.^26,29^ Limitatons on peri-operative mobility were only documented in one study, where cementless THA patients were instructed to partially weight-bear for 3 weeks post-operatively.^27^

Use of thromboprophylaxis around the time of intervention was documented in five studies.^25,27–30^ Two studies used prophylactic low molecular weight heparin (LWMH) only,^27,29^ whilst three used a combination of prophylactic LMWH, fondaparinux, warfarin (INR target 1.85–2.5), and 150–325 mg aspirin.^25,28,30^ Where specified, LWMH dosing was consistent across the patient cohorts.^27,30^ Only two studies documented the duration of thromboprophylaxis given.^25,30^ One initiated prophylaxis within 24 h of surgery and continued therapy for 3 weeks post-operatively^25^; whilst the other started prophylaxis 6 h post-surgery and continued treatment for 2–5 weeks dependent on the operative intervention (TKA vs THA).^30^ The use of mechanical prophylaxis was stated in three studies,^25,28,30^ but only one study included treatment details.^25^ Here, patients were instructed to wear thigh-high compression stockings continuously for at least 3 weeks post-operatively.

Included surgical patients were divided based on the presence or absence of recent flight. The indication for air travel was recorded in five studies.^26,28–31^ Four^26,28–30^ cited travel for the purposes of medical treatment as the primary reason for flying, whilst the remaining study reported cases of employment-related flight.^31^ In five studies air travel occurred post-operatively.^25,26,28,30,31^ In four of these, travel occurred within 45 days of operative procedure,^25,26,28,30^ with a mean time interval of 2–6 days documented in three studies.^26,28,30^ In the remaining study with post-operative air travel, the time interval between the operation and flight was not documented.^31^ The studies either documented a mean duration or distance of travel, with significant variation across different study populations. Five studies included information on travel duration.^25,27,28,30,31^ Three specified a range of travel times from 0.7 to 13.7 h (mean 1.1–4.0 h)^25,28,30^; whilst the other two stated all flights were >4 h.^27,31^ Two studies documented flight distance instead of time duration.^26,29^ One listed a range of distances from 486 to 9684 km (median 1783 km),^26^ while the other described all air travel as >5000 km.^29^

### Rate of VTE

**Figure 1.**
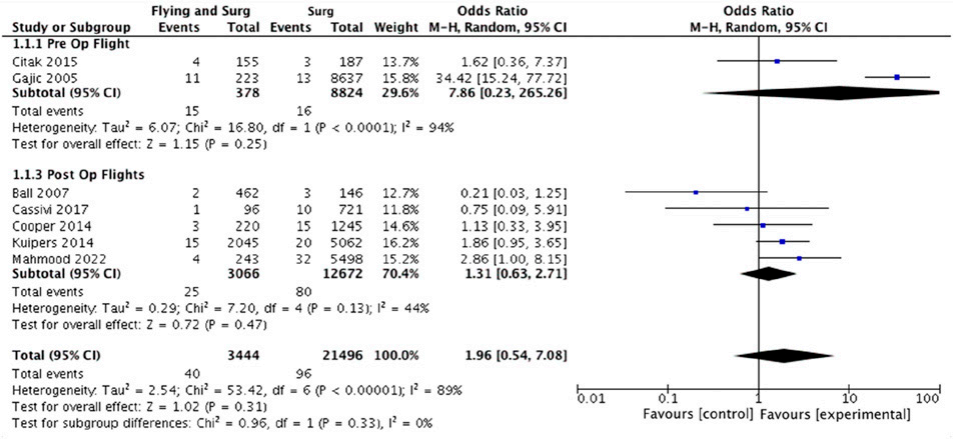
Forest plot demonstrating the overall pooled odds ratio of venous thromboembolism with flying and surgery and subgroup analysis of the pooled odds ratio of venous thromboembolism with pre-operative and post-operative air travel. Abbreviations List: confidence interval (CI); Mantel-Haenszel (M-H).

**Figure 2.**
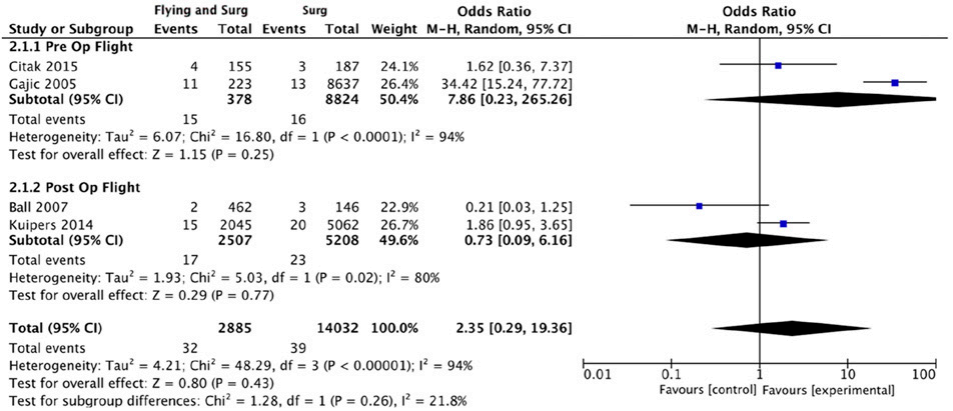
Forest plot demonstrating subgroup analysis of the pooled odds ratio of venous thromboembolism in surgical patients with average flight time >4 h. Abbreviations List: confidence interval (CI); Mantel-Haenszel (M-H).

**Figure 3.**
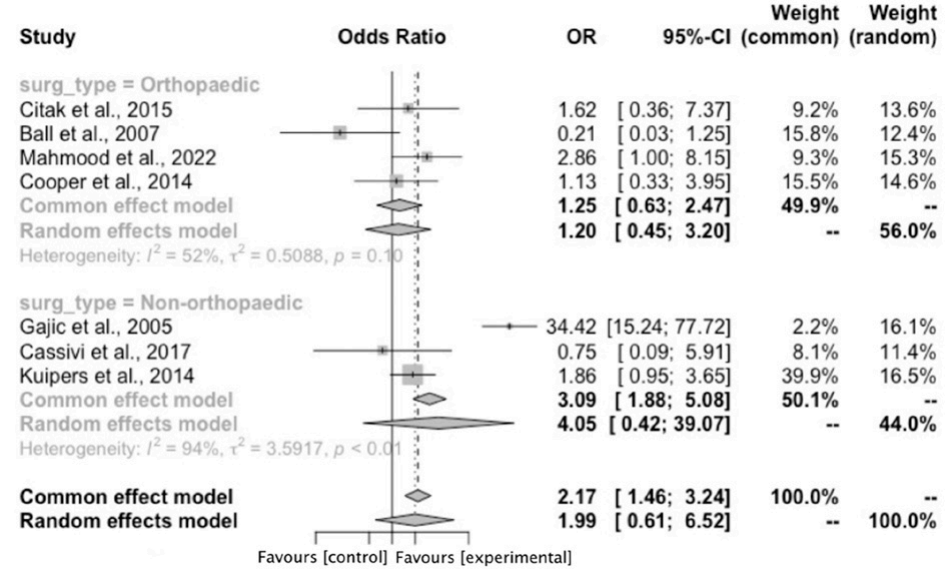
Forest plot demonstrating subgroup analysis of the pooled odds ratio of venous thromboembolism with air travel and patients undergoing high venous thromboembolism risk interventions. Abbreviations List: confidence interval (CI); odds ratio (OR).

The number of symptomatic VTE events were extracted from all included studies. Where recorded,^27,29,31^ diagnosis following symptom onset was confirmed using a combination of computed tomography, angiography, venous ultrasonography and echocardiography. Overall, there were 40 VTE events in 3444 patients in the surgery and flying group versus 96 VTE events in 21,496 patients in the surgery alone group. The pooled odds ratio (OR) of VTE in the flying plus surgery group was 1.96 (95% CI: 0.54–7.08, *p* = .31) (Figure 1). Heterogeneity measured via I^2^ was 89%. Subgroup analysis was undertaken to attempt to explain this elevated heterogeneity (Figure 1). For patients with post-operative air travel, the OR of VTE was 1.31 (95% CI: 0.63–2.71, *p* = .47) (5 studies, 105 events, 15,738 patients, I^2^ = 44%).^25,26,28,30,31^ For surgery and pre-operative air travel, the OR of VTE was 7.86 (95% CI: 0.23–265.26, *p* = .25) (2 studies, 31 events, 9202 patients, I^2^ = 94%).^27,29^ Given previous literature documentation specifying elevated VTE risk only after >4 h of flight time,^15^ subgroup analysis was undertaken of studies with average flight time >4 h^25,27,29,31^ (Figure 2). The OR of VTE in this flying and surgery group was 2.35 (95% CI: 0.29–19.36, *p* = .43) (4 studies, 71 events, 16,917 patients, I^2^ = 94%). Additionally, major lower limb orthopaedic interventions are associated with particularly high VTE risk.^18^ Therefore further subgroup analysis was conducted for studies assessing THA and TKA patients only^25,27,28,30^ (Figure 3). In this high risk flying and surgery group, the OR of VTE was 1.20 (95% CI: 0.45–3.20, *p* = .47) (4 studies, 66 events, 8156 participants, I^2^ = 52%).

Three studies reported DVT incidence specifically.^25,28,30^ A logit transformation was applied, and pooled DVT rate was calculated. For patients with surgery and recent flight, the pooled DVT rate was 0.67 (95% CI: 0.31%–1.51%) (3 studies, 6 events, 925 patients). The pooled DVT rate in patients with surgery alone was 0.45% (95% CI: 0.10%–2.00%) (3 studies, 15 events, 6889 patients).

Three studies additionally reported PE incidence.^25,28,30^ A Freeman–Tukey double-arcsine transformation was applied, and pooled PE rate was calculated. For patients with surgery and recent flight, the pooled PE rate was 0.41% (95% CI: 0.00%–1.29%) (3 studies, 3 events, 925 patients), whilst the pooled PE rate in patients with surgery alone was 0.55% (95% CI: 0.31%–0.86%) (3 studies, 35 events, 6889 patients).

Only two studies collected data on VTE-related mortality and were unsuitable for meta-analysis due to a zero-event rate in one study.^25,29^ The crude mortality rate for surgery and flying was 0.15% (2 studies, 1 event, 685 patients). For patients with surgery only, VTE-related mortality rate was 0.03% (2 studies, 2 events, 8783 patients).

### Risk of bias

Risk of bias was assessed using the ROBINS-I tool (Supplemental Table 2). Due to the non-randomised nature of included studies, there was serious/critical risk of bias found in the majority; particularly regarding selection of study participants, lack of control of confounding variables, assessment of primary outcomes, inconsistency around reporting of attrition and potential for selective reporting of outcomes.

### GRADE assessment

GRADE assessment was performed to assess the quality of evidence supporting the primary outcome. All included studies were observational in nature, making the initial quality of evidence low. Due to the serious risk of bias and imprecision in confidence intervals presented, certainty was downgraded to very low.

## Discussion

This is the first study to pool all published literature on the association between air travel and VTE rate in surgical patients. This has found that both flying and non-flying surgical patients share similar reported pooled rates of VTE. Whilst odds ratios displayed a tendency towards increased VTE risk in the flying cohort, the data failed to reach statistical significance. This holds true when looking at DVT and PE incidence specifically, and is maintained across subgroup analysis of both pre-operative and post-operative air travel, air travel >4 h and high VTE risk surgery.

During full-text screening there was a subgroup of six studies that initially appeared eligible for inclusion, but did not report the primary outcome of VTE rate.^32–37^ They were excluded because they analysed a cohort positively identified with VTE and looked retrospectively to see which patients had both surgery and recent air travel as dual risk factors (Table 2). Interestingly, the majority seem to report both flying and surgery as independent risk factors for developing VTE, and when both exposures co-exist together VTE risk increases. On average, 6% (inter-quartile range: 3–9%) of these pooled VTE patients reported surgery and flying as combined risk factors. However, these studies must be interpreted with caution due to significant flaws in their methodology, particularly regarding retrospective data collection and analysis increasing risk of detection and reporting bias.

**Table 2.** Demographic data for excluded papers analysing the incidence of flying and surgery in venous thromboembolism patients.

Paper	Study design	Number of patients	VTE risk factors accounted for	Surgery type	Use of prophylactic compression	Use of prophylactic anti-coagulation	Flight duration	Flight timing	Interval between flying and surgery	Number of patients with combined flying & surgery risk factors
Arfvidsson 1999	Retrospective cohort study	109 patients with VTE, 25 with recent air travel	BMI, hormone therapy, malignancy, steroid use, chronic disease, smoking, recent trauma, alcohol use	Operations for malignancy	No	No	Range of 5 – 18 h, mean of 9 h	Not documented	Not documented	3
Eklof 1996	Retrospective cohort study	254 patients with VTE, 44 with recent air travel	Hormone therapy, malignancy chronic disease, previous DVT	Surgery +/− femoral catheterisation	Not documented	Only documented use in one patient	Range of 5 – 17 h, mean not documented	Pre-operatively	<21 days	4
Hanslow 2006	Retrospective cohort study	602 patients with recent surgery, 24 patients with VTE	Hormone therapy, chronic disease, previous DVT	Lower limb orthopaedic (foot and ankle surgery)	Not documented	Yes	Not documented	Pre-operatively	<14 days	3
Hughes 2006	Retrospective cohort study	576 patients with VTE, 60 with recent air travel	Previous VTE, family history of VTE, malignancy, hormone therapy, recent trauma, recent travel other than flight, pregnancy	Not documented	Not documented	Not documented	Ranges of <2 h, 2–4 h, 4–10 h, 10–20 h, >20 h; mean not documented	Not documented	<28 days	0
MacCallum 2011	Case – control study	550 patients with VTE, 55 with recent air travel	BMI, previous VTE, duration of inpatient stay	Low risk (day-case), medium risk, and high risk (malignancy/orthopaedic)	Not documented	Not documented	Ranges of 0–4 h, 4–8 h, 8–12 h, >12 h; mean not documented	Not documented	<28 days	Specific number not documented; however paper includes graphical illustration of predicted ORs of VTE in surgical patients with increasing flight duration
Mercer 1998^33^	Nested case control study	134 patients with VTE, 33 with recent air travel	Hormone therapy, malignancy, previous VTE, chronic disease	Lower limb orthopaedic	Not documented	Not documented	>4 h, range not documented	Not documented	Not documented	1

Abbreviations List: ASA, American society of anaesthesiologists physical status classification system; BMI, body mass index; DVT, deep vein thrombosis; INR, international normalised ratio; OR, odds ratio; VTE, venous thromboembolism.

The lack of prospective, randomised studies included in the results introduced significant risk of bias. Comprehensive statistical analysis was performed to attempt to account for multiple confounding variables. Previous literature suggests that long-haul flights (>4 h) confer increased VTE risk.^15,38^ This risk becomes more significant the greater the flight time and period of immobility.^15,39^ In the included studies there was broad variability in duration of travel, ranging from 40 min to 13 h 42 min. For Cooper et al.^28^ and Mahmood et al.,^30^ mean flight time for all patients failed to reach this literature supported benchmark of 4 h. This may have contributed to the lack of significance in VTE rate demonstrated between our two study groups. However, when subgroup analysis was undertaken to exclude all studies with documented travel times <4 h, the lack of significant increase in VTE rate persisted. This suggests that air travel may not confer additional risk for VTE development in surgical patients, regardless of flight time. Additionally, post-operative VTE incidence varies with the type of surgical intervention, with highest risk associated with major lower limb orthopaedic operations, and lowest risk with laparoscopic day case procedures.^18,40^ Only four papers included lower limb orthopaedic interventions (THA and TKA), so subgroup analysis was conducted to assess VTE risk in this high-risk cohort.^25,27,28,30^ This revealed no difference in VTE risk between the flying and non-flying groups, implying that peri-operative air travel does not impact on post-operative VTE incidence, regardless of operation-dependent VTE risk.

For the pooled DVT analysis, high statistical heterogeneity was noted in non-flying surgical patient data from Ball et al.^25^ One explanation for this is unlike other papers, in Ball et al., non-flying surgical patients were exposed to another form of extended travel as a comparator against flying. For other studies, extended travel in the comparator group was either not mentioned or was specifically stated not to have occurred. Therefore, the overall observed DVT rate in the comparator population may be higher than predicted due to confounding immobility during prolonged car and train travel in Ball et al., data, which is a known VTE risk factor.^41,42^

### Limitations

Our analysis is limited by included study design, risk of detection/recall bias, heterogeneity in thromboprophylaxis applied, surgery type and variation in flight duration. VTE risk between patients is very heterogeneous and strong VTE risk factors such as family history and previous VTE were not accounted and controlled for in every study as patients were not formally risk-assessed prospectively. This could have directly impacted effect size, over-estimating VTE rate in the comparator cohort. Most studies collected data via retrospective questionnaires sent out to a cohort of surgical patients over a set period. This introduced significant risk of recall bias, where patients positively identified with VTE may have been more likely to respond to the survey and recall information around the time of their operation, including travel mode and duration. Additionally, studies assessing peri-operative VTE development may have been less likely to report flying as a risk factor if it was found to be non-significant, contributing to reporting bias.

Furthermore, despite thromboprophylaxis being one of the most important factors reducing VTE incidence,^43^ its use across the seven studies was poorly documented. This lack of standardisation is likely to have diminished our estimate of VTE rate, due to potential differential use of thromboprophylaxis across the flying and non-flying groups. Variation in operation specific VTE risk and surgical acuity may have also affected our results, where two studies included medium VTE risk surgery only,^26,29^ and one did not document surgery type at all.^31^ This could have contributed to the broadly similar VTE rates seen across the flying and non-flying cohorts, where patients undergoing lower VTE risk operations are intrinsically less likely to develop VTE post-flight. The most important limiting factor in our analysis was flight time variation. As previously shown, two included studies’ mean flight times failed to reach 4 h^28,30^ – the literature consensus on when air travel starts to influence VTE rate. Additionally, two other papers did not state flight duration at all.^26,29^ This may have impacted our conclusion, reducing estimated VTE rate as for those papers with short flight durations there is unlikely to be associated additional increased VTE risk.

All surgical patients should be prospectively risk assessed and VTE prophylaxis delivered on an individualised basis. Type of surgery and duration of air travel should be accounted for. There is need for future prospective cohort studies exploring the relationship between different subgroups of surgical intervention, air travel >4 h and different thromboprophylaxis regimens. Follow up should be standardised for a minimum of 6 weeks using objective imaging techniques to assess for VTE development.

## Conclusion

The current data suggests that air travel may not confer any additional risk for VTE development in patients undergoing surgery. Whilst there is a tendency for increased VTE risk in the flying population, data failed to reach significance. This conclusion is limited by study design, variation in flight characteristics and thromboprophylaxis, and the impact of other associated VTE risk factors in patients across the included studies. Future research could risk score then propensity match patients during analysis to help reduce the effect of confounding.

## Supplemental Material


Supplemental Material - A systematic review and meta-analysis of venous thromboembolism risk in surgical patients with recent air travel
Supplemental Material for A systematic review and meta-analysis of venous thromboembolism risk in surgical patients with recent air travel by Jessie Shea, Avik Ghosh, Benedict RH Turner, Alun H Davies and Sarah Onida in Journal of Phlebology.

## Data Availability

Data used in construction of this article is available on request. Please contact the corresponding author for more information.*
