# Supplementary material for: A systematic review and meta-analysis of venous thromboembolism risk in surgical patients with recent air travel
Source: Phlebology. 2025 May 14;41(1):5–14. doi: 10.1177/02683555251342912 (PMC12812180; doi:10.1177/02683555251342912)
Supplement: Supplemental Material - A systematic review and meta-analysis of venous thromboembolism risk in surgical patients with recent air travel [file sj-pdf-1-phl-10.1177_02683555251342912.pdf]

SUPPLEMENTARY MATERIAL

Supplementary Figure 1: Preferred reporting items for Systematic Reviews and Meta-Analysis (PRISMA) flow diagram of papers assessing the combined impact of air travel and surgery on venous thromboembolism risk

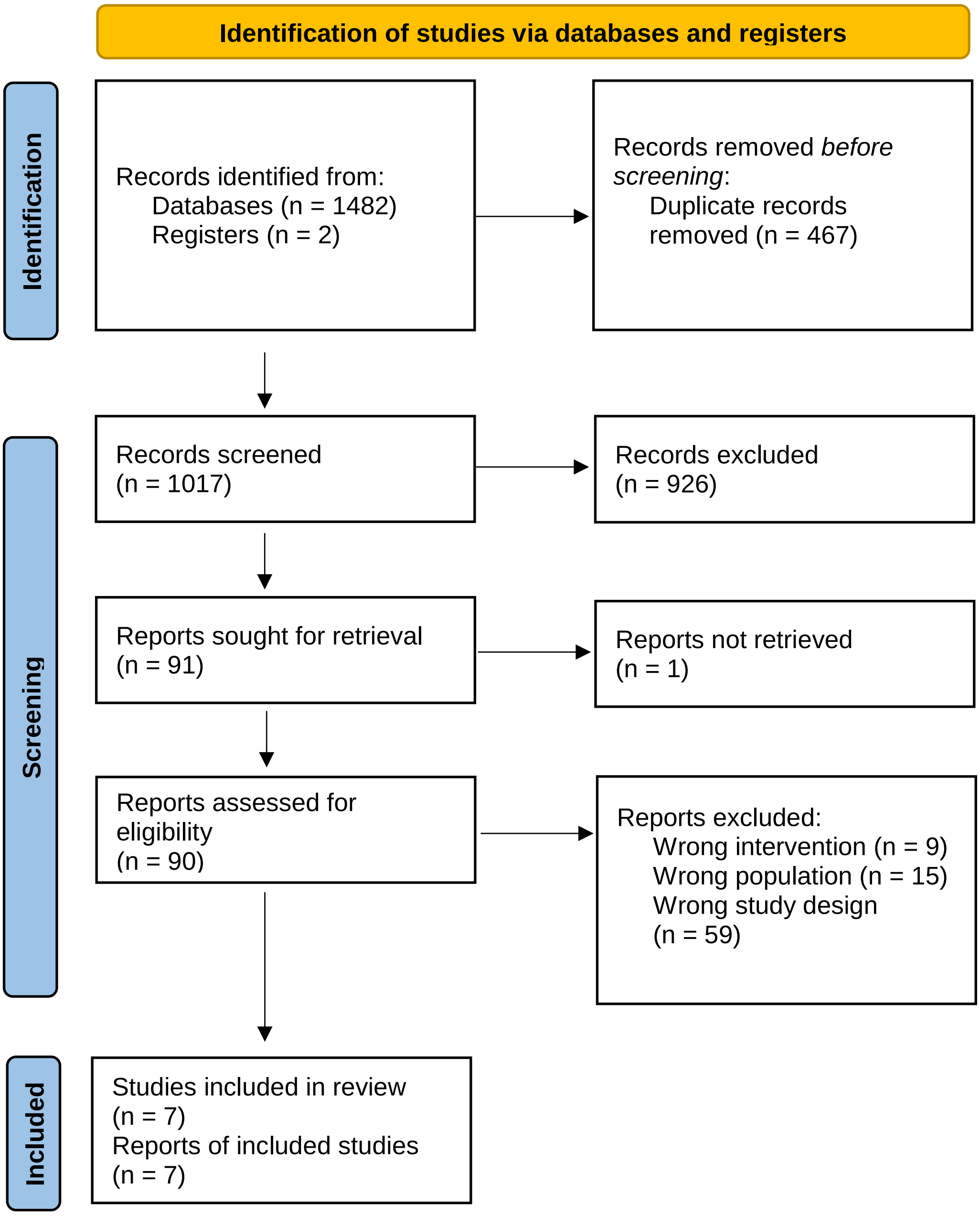

Supplementary Table 1: Search strategy terms used in this systematic review assessing the combined impact of air travel and surgery on venous thromboembolism risk

**Search terms**	
(flying or flight* or airplane* or plane* or aeroplane* or aviation* or aerial navigation* or air travel* or long haul)	
AND	
(venous thromb* or vein thromb* or pulmonary emboli* or lung emboli* or VTE or DVT)	
AND	
(surger* or surgical or operation* or operative or intervention* or post op* or postop* or pre op* or preop* or procedure*) OR (ablation* or radiofreqency or laser or (thermal or nonthermal) or mechanochemical or cyanoacrylate* or CAE or MOCA or sclerotherapy* or UGFS or avulsion or phlebectom*)	
Total number before deduplication (Embase, Medline, Cochrane Library, Scopus)	1482

Supplementary Table 2: Risk of bias assessment using the Risk of Bias in Non-Randomised Studies of Interventions (ROBINS-I) tool of studies assessing the combined impact of air travel and surgery on venous thromboembolism risk

Study	Bias due to confounding	Bias in selection of participants into the study	Bias in classification of interventions	Bias due to deviations from intended interventions	Bias due to missing data	Bias in measurement of outcomes	Bias in selection of the reported result	Overall Bias
Ball 2007	Serious	Low	Moderate	Low	Low	Serious	Moderate	Serious
Cassivi 2017	Moderate	Low	Low	Low	Low	Moderate	Moderate	Moderate
Citak 2015	Moderate	Low	Low	Low	Low	Moderate	Serious	Serious
Cooper 2014	Serious	Low	Low	Low	Low	Serious	Moderate	Serious
Gajic 2005	Serious	Low	Low	Low	Low	Serious	Serious	Serious
Kuipers 2014	Serious	Serious	Low	Low	Moderate	Moderate	Critical	Critical
Mahmood 2022	Moderate	Low	Low	Low	Low	Moderate	Low	Moderate
